# Physician’s Visiting List, Diary, &c.

**Published:** 1857-11

**Authors:** 


ART. XIII.—*The Physician's Visiting List, Diary, and Book of Engagements for* 1858. Philadelphia: Lindsay & Blakiston.

THIS little volume, put up in pocket-book form, comprises an almanac, table of signs, an account of poisons and their antidotes, a table for calculating the period of utero-gestation, and blank leaves for visiting list, and memoranda for every day in the year. The physician cannot fail to find it useful and convenient.

